# Precision Medicine for Brain Disorders: New and Emerging Approaches

**DOI:** 10.3390/jpm13050872

**Published:** 2023-05-22

**Authors:** Zack Y. Shan, Jim Lagopoulos

**Affiliations:** Thompson Institute, University of the Sunshine Coast, Birtinya, QLD 4575, Australia

The brain is the most complex organ in the human body, making it susceptible to many abnormalities. Brain disorders include but are not limited to neurodegenerative brain diseases (Alzheimer’s disease, Parkinson’s disease, and amyotrophic lateral sclerosis), mental illness (anxiety, bipolar disorder, depression, post-traumatic stress disorder, and schizophrenia), stroke, epilepsy, neurodevelopmental disorders (attention deficit hyperactivity disorder, autism spectrum disorder, and dyslexia), autoimmune brain diseases (multiple sclerosis and encephalitis), traumatic brain injuries and concussion, brain tumours (astrocytoma, glioblastoma, meningioma,), meningitis, myalgic encephalomyelitis/chronic fatigue syndrome, and hundreds of other rare brain diseases.

Brain disorders are the foremost contributor to disability and the second most common cause of mortality globally [1]. Population growth and increases in ageing, have contributed to a rise in the prevalence of brain disorders that cause disability, particularly with increasing age. The corollary of this increase in brain disorders is that governments will encounter mounting demands for new treatments, rehabilitation, and support services [1]. Therefore, managing brain disorders represents a formidable challenge in contemporary times. In this regard, many are looking to neuroscience for answers to these vexing questions and to bring about a paradigm shift in healthcare, comparable to the transformative impact of the past two centuries advancements in vaccines and antibiotics. Although human brains share common structures and functions, each is unique biologically [2] as evidenced by the fundamental core of one’s personality, individuality, and cognitive abilities. Thus, not surprisingly, the uniqueness of brain disorders is greater than other organ diseases. Consequently, the hitherto ‘biological’ conceptualisation of a molecular and cellular mechanism capturing disease processes for all individuals has been ineffective [3,4,5].

Recently, precision medicine has gained interest in many brain disorders, including stroke [3], psychiatric disorders [4], Alzheimer’s disease [5], and epilepsy [6,7]. Precision medicine is a medical model in which clinical interventions, therapeutic regimens, diagnostic techniques, and medical products are custom-tailored to subgroups of patients rather than relying on a homogeneous “one-drug-fits-all” approach [8]. This editorial paper calls for more targeted research towards precision medicine of brain disorders, which is built on four pillars: (i) identification of biomarkers, (ii) systems medicine, (iii) digital health technologies, and (iv) data science [9].

Biomarkers: we here consider biomarkers, according to World Health Organization (WHO), as “any substance, structure, or process that can be measured in the body or its products and influence or predict the incidence of outcome or disease” [10]. Biomarker technologies for brain disorders include omics (genomics, transcriptomics, proteomics, metabolomics, epigenomics, microbiomics, cytomics, and lipidomics) and neuroimaging (structural MRI, functional MRI, diffusion tensor imaging, positron emission tomography, magnetoencephalography, electroencephalography, optical imaging).

Systems medicine examines the interplay among biochemical, physiological, and environmental factors in the human body as constituents of a cohesive entity. Systems medicine conceptualises physiological processes and disease evolution in a bottom-up strategy using omics. For example, genomic, tissue-level, and single-cell transcriptomics, as well as epigenetic data, have been integrated to discern gene regulatory networks in the brain, thus enabling the prognostication of endo- and syndromic phenotypes associated with psychiatric disorders [11]. An alternative top-down approach to systems medicine involves identifying biomarkers as a starting point and subsequently identifying the necessary molecular conditions associated with the corresponding brain function. Neuroimaging-guided subtyping of psychiatric disorders has emerged recently (see Scangos et al. [4] for a review), although the molecular underpinning remains elusive.

Digital technologies: in the past decade, the rapid advancement of computer science has resulted in the swift development of digital technologies such as electronic health/medical records, wearable smart devices, and smartphones. Brain disorders are tightly related to physiological systems and functional domains such as sleep, circadian rhythm, complex behaviours, and social interactions. As such, digital technologies open new possibilities including the potential for longitudinal monitoring of physiological data in situ.

Data science: the development of biomarkers and digital technologies has resulted in large amounts of data. Big data analysis poses challenges due to its immense quantity, heterogeneous nature, issues with harmonisation and complex structure, which render traditional statistical methods inadequate. Moreover, traditional statistical techniques prioritise hypothesis testing over prediction. In contrast, machine learning-based computational models offer promising alternatives, as they can generate clinically meaningful insights from sparse and noisy multidimensional data originating from various sources [12].

Precision medicine holds great potential for generating mechanistically guided treatments for brain disorders, even accounting for the brain’s complexity and individuality. However, despite significant progress in other areas, precision medicine in the context of neurological and psychiatric diseases has yet to be fully realised. At this critical juncture, we invite contributions related to any of the four aspects of precision medicine related to the brain.
